# Prospective Validation of Cessation of Contact Precautions for Extended-Spectrum β-Lactamase–Producing *Escherichia coli*^1^

**DOI:** 10.3201/eid2206.150554

**Published:** 2016-06

**Authors:** Sarah Tschudin-Sutter, Reno Frei, Friedbert Schwahn, Milanka Tomic, Martin Conzelmann, Anne Stranden, Andreas F. Widmer

**Affiliations:** University Hospital Basel, Basel, Switzerland (S. Tschudin-Sutter, R. Frei, F. Schwahn, A. Stranden, A.F. Widmer);; Felix Platter Hospital, Basel (M. Tomic, M. Conzelmann)

**Keywords:** extended-spectrum beta-lactamases, ESBL, Enterobacteriaceae, transmission, contact precautions, ESBL-producing E. coli, Escherichia coli, infection control measures, nosocomial spread, Switzerland, bacteria

## Abstract

After contact precautions were discontinued, we determined nosocomial transmission of extended-spectrum β-lactamase (ESBL)–producing *Escherichia coli* by screening hospital patients who shared rooms with ESBL-producing *E. coli*–infected or –colonized patients. Transmission rates were 2.6% and 8.8% at an acute-care and a geriatric/rehabilitation hospital, respectively. Prolonged contact was associated with increased transmission.

The rapid increase of extended-spectrum β-lactamase (ESBL)–producing *Enterobacteriaceae* has challenged healthcare facilities worldwide regarding implementation of effective infection-control measures to limit further nosocomial spread (*1*). The benefits of routine enforcement of contact precautions must be balanced against additional costs, impediments to patient care, and exposure to ESBL-producing *E. coli* outside healthcare institutions.

At the University Hospital Basel (UHB), a university-affiliated tertiary care center in Basel, Switzerland, transmission rates of ESBL-producing *Escherichia coli* are low in contact patients exposed to patients colonized or infected with ESBL-producing *E. coli*. This low transmission rate challenges the routine use of contact precautions in nonepidemic settings (*2*). Based on our findings and recent data suggesting that ESBL-producing *E. coli* is predominantly acquired in the community (*3*), we abandoned contact precautions for patients infected or colonized with ESBL-producing *E. coli* at the UHB and an affiliated long-term care center, Felix Platter Hospital (FPH), in Basel. To validate this practice, we screened all patients who shared a hospital room with a patient with ESBL-producing *E. coli* to determine transmission rates.

## The Study

UHB is an acute-care hospital with 735 beds, of which 8.7% are in rooms with 4 beds and the remaining are in rooms with 1–2 beds. FPH is a university-affiliated geriatric and rehabilitation center with 320 beds, of which 47.5% are in rooms with 4 beds and 52.5% are in rooms with 1–2 beds. In both facilities, the average distance between beds is 2 m. The 2 institutions share an infection-control team and microbiology laboratory. The study was approved by the local ethics committee as part of the quality assurance program; informed consent was waived.

FPH and UHB abandoned routine contact precautions for patients infected or colonized with ESBL-producing *E. coli* beginning January and June 2012, respectively; patients were included in this study through December 2013. We defined index patients as patients colonized or infected with an ESBL-producing *E. coli* in any specimen from any body site and contact patients as patients hospitalized for at least 24 hours in the same room as an index patient. Contact time was defined as the time index and contact patients shared a room before the contact patient was screened. Contact patients were prospectively screened once before discharge by swab sampling of the rectum and any open wounds or drainage sites; if Foley catheters were used, urine was also sampled and cultured. Transmission was considered to have occurred if ESBL screening results for the contact patient were positive and molecular typing by pulsed-field gel electrophoresis (PFGE) showed that the strain shared identity with the strain of the index patient.

We used standard culture methods with chromogenic medium (chromID ESBL; bioMérieux, Marcy l’Etoile, France) to detect ESBL-producing *E. coli*. We performed routine identification and susceptibility testing using the Vitek 2 System (bioMérieux, Durham, NC, USA) with cefpodoxime, ceftriaxone, and ceftazidime. We confirmed positive results by using Etest strips (bioMérieux, Marcy l’Etoile) containing cefotaxime or ceftazidime, with and without clavulanic acid. We used molecular typing by PFGE to determine the identity of strains.

We used the Fisher exact test and the Mann-Whitney U test for univariable comparisons. Logistic regression was performed to calculate odds ratios for transmission. Two-sided p<0.05 was considered significant.

During the study period, 231 contact patients (151 from UHB, and 80 from FPH) were exposed to 211 index patients (178 from UHB, 33 from FPH). Contact patients were screened for ESBL-producing *E. coli* after a median contact time of 4 (interquartile range 3–6) days at UHB and 15 (interquartile range 9–23) days at FPH.

**Figure 1:**
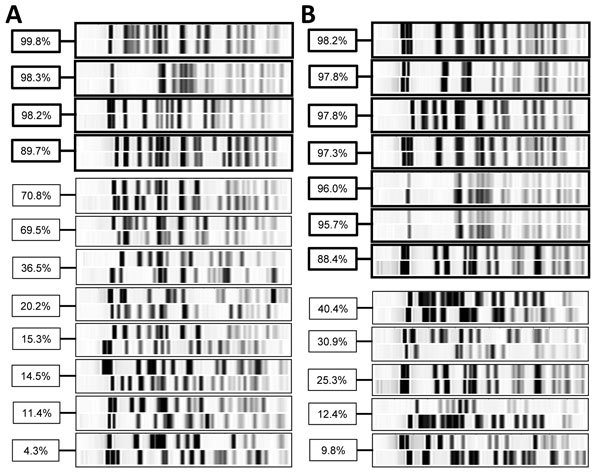
Pulsed-field gel electrophoresis results for *Escherichia coli* samples from A) index and B) contact patients who shared rooms for at least 24 hours in an acute-care hospital or a geriatric/rehabilitation center, Basel, Switzerland. Thick black outlining indicates results for patient pairs with extended-spectrum β-lactamase–producing *E. coli* transmission. FPH, Felix Platter Hospital; UHB, University Hospital Basel.

**Figure 2:**
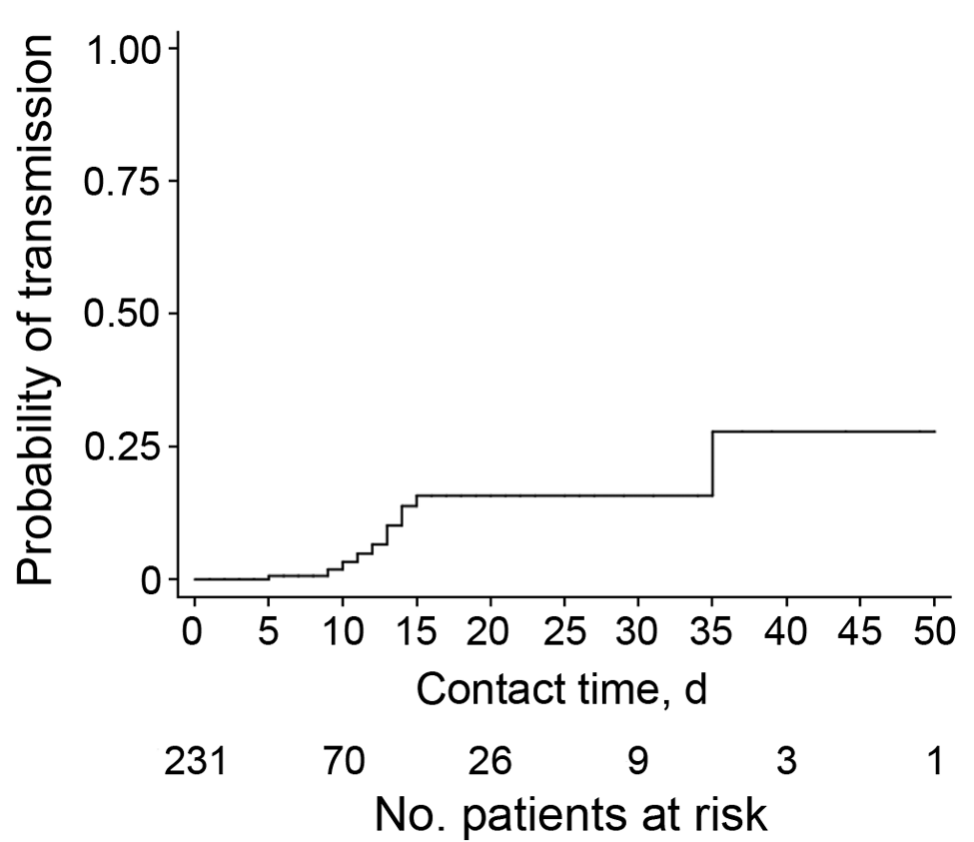
Transmission of extended-spectrum β-lactamase–producing *Escherichia coli* over contact time among index and contact patients who shared rooms for at least 24 hours in an acute-care hospital or a geriatric/rehabilitation center, Basel, Switzerland.

We recovered ESBL-producing *E. coli* from 24 contact patients (12 from each institution) and confirmed strain identity for 11, accounting for an overall transmission rate of 4.8% (11/231) (Figure 1). Transmission occurred in 2.6% (4/151) of contacts at UHB and 8.8% (7/80) at FPH (p = 0.052). We found no differences between contact patients with and without transmission of ESBL-producing *E. coli* in regard to baseline characteristics; use of antimicrobial drugs; or exposure to index patients, except for contact time, which was longer for patients with transmission (Table). Exposure to an index patient for >5 days was associated with increased odds for transmission (odds ratio 10.18, 95% CI 1.28–80.91; p = 0.028) (Figure 2).

**Table:** Characteristics and exposures for hospitalized contact patients with and without transmission of ESBL-producing *Escherichia coli* from index patients, Basel, Switzerland*

Patient characteristics and exposures	Contact patients with transmission of ESBL-producing *E. coli*, n = 11†	Contact patients without transmission of ESBL-producing *E. coli*, n = 220†	p value
Contact patient characteristics			
Age, y, median (IQR)	81 (77–82)	75 (64–82)	0.153
Charlson Comorbidity Index, median (IQR)	2 (1–4)	2 (1–3)	0.399
Contact time, d, median (IQR)	13 (10–15)	8 (5–12)	**0.006**
Intensive care unit stay	0	54 (24.8)	0.122
Received any antimicrobial drug	4 (36.4)	93 (42.3)	0.765
Received systemic antimicrobial drugs with activity against ESBL *E. coli*	1 (9.1)	19 (8.6)	1.000
Index patient characteristics			
Age of index patient, y, median (IQR)	79 (64–87)	73 (62–80)	0.175
Charlson Comorbidity Index, median (IQR)	2 (1–3)	2 (1–3)	0.572
Infected with ESBL-producing *E. coli*	6 (54.6)	84 (38.2)	0.346
ESBL-producing *E. coli* infection			
Bloodstream	0	3 (1.4)‡	1.000
Urinary tract	5 (45.5)	68 (30.9)	0.330
Respiratory tract	1 (9.1)	10 (4.6)	0.422
Surgical site	0	6 (2.7)	1.000
Colonized with ESBL *E. coli*	5 (45.4)	136 (61.8)	0.346
Received systemic antimicrobial drugs with activity against ESBL *E. coli*	6 (54.6)	84 (38.2)	0.346

*Bold indicates significance. Contact patient exposures occurred through the sharing of a room for at least 24 hours with an ESBL-producing *E. coli*–infected or colonized index patient in an acute-care hospital or a geriatric/rehabilitation center. ESBL, extended-spectrum β-lactamase; IQR, interquartile range. †Values are no. (%) patients except as indicated. ‡All patients with bloodstream infections had urinary tract infections.

## Conclusions

After contact precautions for ESBL-producing *E. coli* were discontinued at the 2 hospitals in this study, transmissions occurred in 2.6% of contact patients at UHB and in 8.8% of contact patients at FPH. Transmissions were associated with duration of hospitalization in the same room as an index patient. At UHB, the rate of transmissions was similar to that reported during the period before discontinuation of contact precaution measures (1.5%) (*2*). At other Swiss acute-care hospitals, ESBL-producing *E. coli* transmission has affected 4.5% of all contact patients (*3*), and transmission of all ESBL-producing *Enterobacteriaceae* has affected 2.8%, despite implementation of contact precautions (*4*). The proportion of contact patients with transmission at FPH (8.8%) compares well with the proportion reported from similar settings (6.5%) (*5*).

Our finding that the transmission rate at the acute-care hospital was similar before and after discontinuation of contact precaution measures may be explained by high adherence to standard precautions (*6*), especially hand hygiene, for which compliance exceeded 90% (*7*), and the mainly short-term hospitalizations (<5 days). Thus, these findings may not be generalizable to other settings, especially when longer hospitalization is required, as is the case in geriatric/rehabilitation centers. Other factors may also have influenced transmission rates in our study, impeding generalizability of the findings to other countries. For example, the European Surveillance of Antimicrobial Consumption Network (http://ecdc.europa.eu/en/healthtopics/antimicrobial_resistance/esac-net-database/Pages/database.aspx) reports that antimicrobial drug use in hospitals in Switzerland (1.9 defined daily doses/1,000 inhabitants/day) is lower than that in hospitals in other European countries (mean of 2.0 defined daily doses/1,000 inhabitants/day). Furthermore, the incidence of ESBL-producing *E. coli* may be lower in Switzerland than in other European countries (*8*), as suggested by a lower proportion (8.2%) of third-generation cephalosporin-resistant *E. coli* among invasive isolates in Switzerland as compared with those in other European countries (http://www.anresis.ch/index.php/anresisch-data-de.html).

Person-to-person transmission may play a substantial role in sustaining the global ESBL epidemic. In nursing homes, ESBL-producing *E. coli* isolates from residents living in adjacent rooms were found to be closely genetically related (*9*), and high ESBL-producing *E. coli* transmission rates (23%) have been reported in households (*3*), supporting our results that sustained contact over longer periods may facilitate transmission. Furthermore, patients hospitalized in the FPH may require more care, resulting in increased contact between healthcare workers and patients, possibly facilitating transmission (*5*).

In our study, contacts were screened only once before discharge, long-term surveillance for acquisition was not performed, and preenrichment of rectal swab samples was not conducted, all of which may have led to an underestimation of ESBL-producing *E. coli* cases. However, the circulation of ESBL-producing clones in the community may have resulted in an overestimation of transmission; before hospitalization, contact patients may have been colonized with strains in the community identical to those of index patients with whom they eventually shared a room. We acknowledge that our study lacks the robustness of a cluster-randomized trial to evaluate the effect of contact precautions on ESBL-producing *E. coli* transmission. However, we found that, when exposure times are short and adherence to standard precautions is high, the discontinuance of contact precautions for ESBL-producing *E. coli* in healthcare settings results in transmission rates similar to those observed when contact precautions are used.
